# Predictors of Exercise-Induced Pulmonary Hypertension in Patients with Asymptomatic Degenerative Mitral Regurgitation: Mechanistic Insights from 2D Speckle-Tracking Echocardiography

**DOI:** 10.1038/srep40008

**Published:** 2017-01-10

**Authors:** Ryo Kamijima, Kengo Suzuki, Masaki Izumo, Shingo Kuwata, Kei Mizukoshi, Manabu Takai, Seisyou Kou, Akio Hayashi, Keisuke Kida, Tomoo Harada, Yoshihiro J. Akashi

**Affiliations:** 1 Division of Cardiology, Department of Internal Medicine, St. Marianna University School of Medicine, Kawasaki, Japan

## Abstract

Presence of exercise-induced pulmonary hypertension (EIPH) in asymptomatic degenerative mitral regurgitation (DMR) determines prognosis. This study aimed to elucidate the mechanism and predictors of EIPH in asymptomatic DMR. Ninety-one consecutive asymptomatic patients with DMR who underwent exercise stress echocardiography were prospectively included. We obtained various conventional echocardiographic parameters at rest and during peak exercise, as well as left atrial (LA) function at rest using 2-dimensional speckle-tracking analysis. The 25 patients (33.3%) with EIPH were significantly older and had a greater ratio of mitral peak velocity of early filling to early diastolic mitral annular velocity during peak exercise than those without EIPH. LA strain (LAS)-s and LAS-e, indices of LA reservoir and conduit function, respectively, were significantly lower in those with EIPH than in those without EIPH. Multivariate analysis indicated that LAS-s was the only resting echocardiographic parameter that independently predicted EIPH, with a cut-off value of 26.9%. Furthermore, Kaplan-Meier curve analysis showed that symptom-free survival was markedly lower among those with reduced LAS-s. In conclusion, decreased LA reservoir function contributes to EIPH, and LAS-s at rest is a useful indicator for predicting EIPH in asymptomatic patients with DMR.

Mitral valve (MV) prolapse (MVP) is a common disorder affecting 2 to 3% of the general population^1^,^2^. Even among patients with severe degenerative mitral regurgitation (DMR), determining optimal timing of surgical treatment is extremely difficult and controversial. Current guidelines recommend MV surgery based on symptomatic status^3^,^4^. However, the postoperative prognosis of DMR depends heavily on presence and extent of preoperative symptoms; prognosis worsens after surgery while a patient is symptomatic compared with while asymptomatic^5^,^6^. In other words, it is essential to predict development of symptoms and perform surgery while patients are still asymptomatic, and exercise stress tests have been considered useful in predicting development of symptoms.

Among asymptomatic patients with DMR, it has been reported that symptom-free survival is extremely poor in those exhibiting exercise-induced pulmonary hypertension (EIPH)^7^. Various factors have been reported as possible mechanisms of pulmonary hypertension (PH) onset in patients with mitral regurgitation (MR), including elevated left ventricular (LV) end-diastolic pressure due to systolic and diastolic dysfunction, elevated left atrial (LA) pressure due to decreased LA compliance, alveolar capillary stress failure, dysfunctional vascular smooth muscle reactivity, distal pulmonary arteriolar hypertrophy, and neointimal proliferation^8^. However, it remains unclear which of these factors contributes most to occurrence of EIPH. Furthermore, facilities that can perform exercise echocardiography are limited by issues related to cost and equipment. Hence, the ability to predict EIPH at rest has important clinical implications. Here, we focused on the left atrium because LA function has been reported as a prognostic factor in various diseases in recent studies^9^,^10^,^11^,^12^,^13^,^14^,^15^. Therefore, the aim of the present study was to elucidate the mechanism and echocardiographic predictors of EIPH in asymptomatic DMR.

## Methods

### Study subjects

In this prospective study, we recruited 91 consecutive asymptomatic patients with DMR with maintained LV systolic function (LV end-systolic diameter <45 mm and LV ejection fraction [LVEF] >60%) and moderate or greater MR (effective regurgitant orifice area (EROA) >0.20 cm^2^ or regurgitant volume [RV] >30 mL) who underwent exercise stress echocardiography at our facility from October 2011 to December 2014. When selecting participants, asymptomatic patients were defined as those who did not exhibit specific symptoms of heart failure with a New York Heart Association functional classification of II or below. Experienced cardiologists conducted interviews with the patients and confirmed medical history and presence of current symptoms. Of these patients, we excluded those with resting PH, defined as pulmonary artery systolic pressure (PASP) ≥50 mmHg according to guidelines^3^ (n = 2); combined valvular heart disease, such as mitral stenosis and aortic valve disease (n = 3); persistent atrial fibrillation (n = 4); those in whom enforcement of exercise proved difficult (n = 3); and those whose imaging results could not be analyzed due to poor image quality (n = 4). Ultimately, 75 patients were registered.

### Conventional echocardiography

Echocardiography was performed in the left lateral decubitus position using a commercially available system (Vivid E9; GE Vingmed, Milwaukee, WI, USA). Images were obtained using a 3.5-MHz transducer in the parasternal and apical views (long-axis, 2- and 4-chamber views). Two-dimensional echocardiography measured each chamber’s dimension, volume, and wall thickness according to the recommendations of the American Society of Echocardiography^16^. LVEF was calculated according to the biplane Simpson’s method in apical 2 and 4-chamber views. Ratio of early (E) to late (A) transmitral velocities (E/A) and deceleration time of E velocity were obtained using pulsed-wave Doppler in the apical 4-chamber view. E’ was measured at the septal mitral annulus in the apical 4-chamber view by placing the sample volume at a sample size of 8 mm. The ratio of mitral peak velocity of early filling (E) to early diastolic mitral annular velocity (E’) (E/E’) was measured to estimate LV filling pressure^17^. MR severity was measured using the doppler volumetric method^18^ and proximal isovelocity surface area method^19^. When both methods could be obtained, the results were averaged, both at rest and during peak exercise. Stroke volume (SV) was calculated by multiplying LV outflow tract (LVOT) area by LVOT velocity-time integral measured using pulsed-wave doppler. Then, cardiac output (CO) was calculated as SV × heart rate. PASP was estimated based on the tricuspid regurgitation jet according to the simplified Bernoulli equation (PASP = 4 × v^2^ + right atrial pressure), in which “v” is peak velocity of the tricuspid regurgitation jet (m/s), and right atrial pressure is estimated from the diameter and breath-induced variability of the inferior vena cava^20^. These conventional echocardiographic parameters were collected at rest and during peak exercise, respectively.

### 2D speckle-tracking analysis

**Figure 1:**
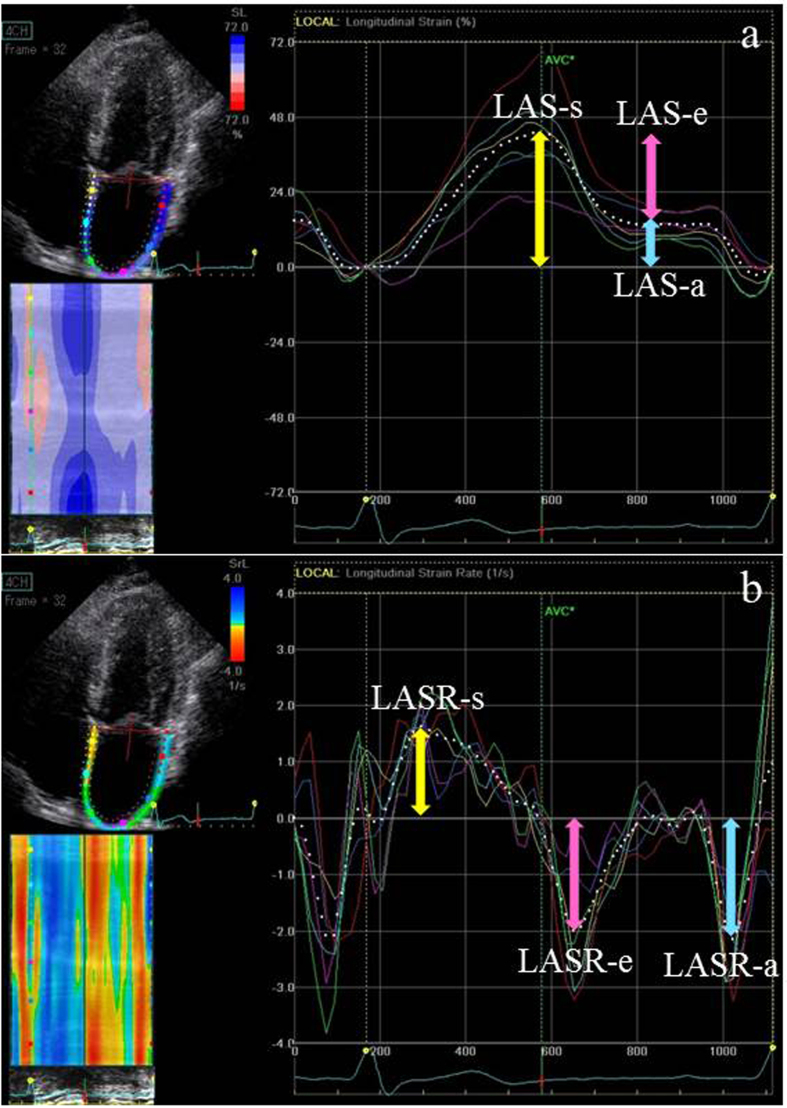
(**a**) Four-chamber left atrial strains (LASs). (**b**) Four-chamber LAS rates (LASRs). The white dotted line represents the mean value of the tracked LA segments. Reservoir (r), conduit (e), and contractile (a) strains are represented by color-coded arrows (yellow, pink, and blue, respectively). Measurements were averaged over 4- and 2-chamber views. In this patient, reservoir, conduit, and contractile strains and strain rates were 41.1%, −25.6%, and −15.5%, and 1.68 s^−1^, −2.0 s^−1^, and −2.2 s^−1^, respectively.

LA strain (LAS) and LA strain rate (LASR) were assessed using 2-dimensional (2D) speckle-tracking analysis with QRS onset as the reference point by applying a commercially available LV strain software package to the left atrium (EchoPAC, version 12; GE Vingmed, Milwaukee, WI, USA)^21^. In summary, the region of interest was adjusted to include the LA myocardium in the apical 4- and 2-chamber views that included both the left atrium and left ventricle. Manual correction was performed to optimize tracking results, if needed. LAS and LASR were calculated as the average value of the 4- and 2-chamber views, as described in Fig. 1. As previously reported, LA reservoir function was calculated as peak systolic LAS-s, whereas LA contractile function (LAS-e) was measured as LAS at QRS wave onset on electrocardiography. Finally, LA conduit function (LAS-a) was assessed as the difference between LAS-s and LAS-e. In addition, LASR-s was measured as the peak systolic positive value; LASR-e, as the early diastolic negative peak value; and LASR-a, as the late diastolic negative peak value. For simplicity, absolute deformation values are reported as positive numbers^22^. In this study, we obtained LA function using 2D speckle-tracking analysis at rest only. In addition, the relationship between LA size and function was evaluated, with LA enlargement defined as LA diameter (LAD) ≥55 mm or LA volume index (LAVI) ≥60 mL/m^2^, similar to a previous study^23^.

### Exercise echocardiography

All patients underwent echocardiography at rest to collect conventional parameters. Then, they also underwent a symptom-limited graded cycle ergometer exercise test in a semisupine position on a tilting exercise table (ergometer and tilt table 750EC; Lode) for continuous 2D echocardiography. After a 3-min workload at 25 watts (W), intensity was increased by 25 W every 3 min. The obtained data were stored digitally. Electrocardiograms, blood pressure, and heart rate were recorded every min. According to guidelines^3^, EIPH was defined as PASP ≥60 mmHg. Criteria for halting the test were development of chest pain, severe dyspnea, severe fatigue, a sustained blood pressure drop, sustained ventricular tachycardia, short runs of 3 or more ventricular premature contractions, pallor, and/or dizziness.

### Symptom-free survival

Follow-up was performed according to current guidelines^3^,^4^. Patients were classified as symptomatic when shortness of breath, dizziness, or syncope with exertion was identified during the follow-up period of 44 ± 21 months. Physical examination and echocardiography were performed by experienced cardiologists, and symptomatic status was carefully assessed.

### Statistical analysis

Continuous variables are presented as mean ± standard deviation (SD), while categorical variables are presented as number (percentage). The unpaired Student’s *t* test and χ^2^ test were used to compare variables between the EIPH and non-EIPH groups, as determined by exercise PASP. Associations of exercise PASP with several conventional echocardiographic parameters were investigated using Pearson’s correlation analysis. Multiple linear regression analysis was performed to evaluate the associations of exercise PASP with clinical and echocardiographic parameters at rest and peak exercise. Factors found to be significant in uni- and multivariate analyses were evaluated using receiver operating characteristic (ROC) curves. Then, sensitivity, specificity, positive predictive value, and negative predictive value for predicting occurrence of EIPH were determined for various cut-off values. Kaplan-Meier curves were constructed to explore differences in symptom-free survival among patient subgroups, stratified according to presence of EIPH or LAS using the ROC curve–based cut-off value, and compared using the log-rank test. Statistical analyses were performed using JMP 10 (SAS Institute Inc., Cary, NC, USA), with statistical significance set at P < 0.05.

The authors had full access to and took full responsibility for the integrity of the data. All authors have read and agree to the manuscript as written.

### Ethics

This study was performed in accordance with the ethical principles set forth in the Declaration of Helsinki, and the study protocol was approved by the Institutional Committee on Human Research of St. Marianna University School of Medicine, Kanagawa, Japan (No. 1288). Written informed consent was obtained from all patients prior to their enrollment.

## Results

### Patient characteristics and echocardiographic parameters

Patients’ clinical features are shown in Table 1. Mean age was 59.1 ± 13.1 years, and 74.7% were men. Anterior and posterior leaflet prolapse were confirmed in 29.3% and 64.0% of patients, respectively. Of the study patients, 25 (33.3%) were assigned to the EIPH group. Although there were no significant differences between groups in terms of sex, body mass index, heart rate, blood pressure, or log of brain natriuretic peptide concentration, the EIPH group was significantly older than the non-EIPH group (64.0 ± 11.2 years vs. 56.6 ± 13.4 years, P = 0.01). In addition, as hypertension was more prevalent in the EIPH group, introduction of calcium channel–blocking oral medication was significantly higher in the EIPH group (44.0% vs. 12.0%, P = 0.002). Causes of MR were similar between groups. Echocardiographic data at rest and during exercise are displayed in Table 2. EROA and RV were 0.41 ± 0.12 cm^2^ and 62.0 ± 18.1 mL, respectively. We found no differences in conventional echocardiographic parameters at rest between groups. During exercise, LVEF increased significantly (+5.9% ± 0.8%, P < 0.001). As expected, LV stroke volume and cardiac output also increased significantly from rest to peak exercise (+3.5 ± 1.1 mL, P = 0.002; +4.1 ± 0.2 L, P < 0.001, respectively). Similarly, EROA also increased from rest to peak exercise (+0.08 ± 0.01 mm^2^, P < 0.001). Twenty-seven (67.5%) of 40 patients with moderate MR developed severe MR by exercise. However, no differences in MR severity, SV, or CO during exercise were observed between groups. On the other hand, the EIPH group had a significantly higher E/E’ than the non-EIPH group (13.7 ± 4.6 vs. 11.1 ± 3.4, P = 0.01).

### LA volume and function

Various LA measurement values are displayed in Table 3. The groups did not differ significantly with regard to LAD or LAVI. However, LAS-s, LASR-s, LAS-e, and LASR-e were significantly lower in the EIPH group (LAS-s: 22.9 ± 5.5 vs. 32.4 ± 6.8, P < 0.001; LASR-s: 1.11 ± 0.26 vs. 1.42 ± 0.33, P < 0.001; LAS-e: 14.8 ± 5.4 vs. 19.9 ± 6.8, P = 0.003; LASR-e: 1.13 ± 0.29 vs. 1.36 ± 0.36, P = 0.003).

**Figure 2:**
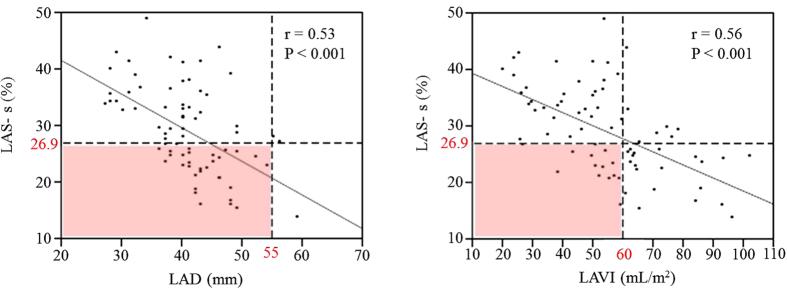
Correlations of left atrial strain (LAS)-s with LA diameter (LAD) and LA volume index (LAVI). LAS-s ≤26.9% (associated with estimated exercise-induced pulmonary hypertension) was present in 44 and 29% of patients despite LAD <55 mm and LAVI <60 mL/m^2^, respectively (pink squares).

**Figure 3:**
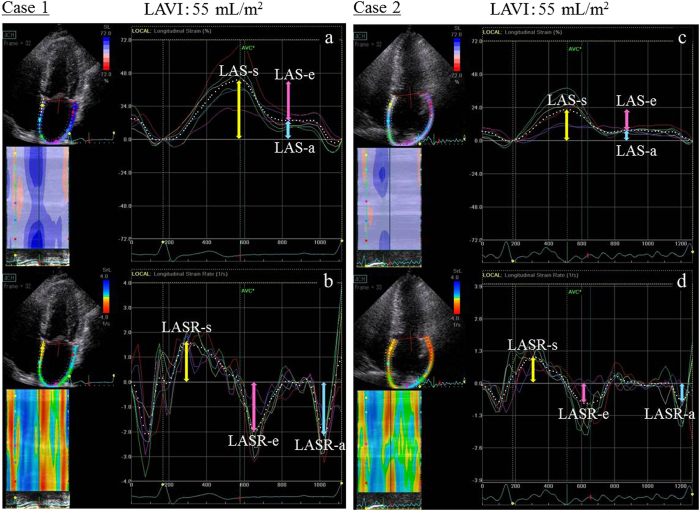
Although cases 1 and 2 had a similar left atrial volume index (LAVI, approximately 55 mL/m^2^), LA function, as indicated by 2D speckle-tracking echocardiography, was significantly different. In case 1, reservoir (r), conduit (e), and contractile (**a**) strains and strain rates were 41.1%, −25.6%, and −15.5%, and 1.68 s^−1^, −2.0 s^−1^, and −2.2 s^−1^, respectively. On the other hand, case 2 had lower reservoir, conduit, and contractile strains and strain rates (22.1%, −13.9%, and −8.2%, and 1.02 s^−1^, −0.70 s^−1^, and −0.82 s^−1^, respectively). Abbreviations: LAS, left atrial strain; LASR, left atrial strain rate.

In addition, there was a negative correlation between LA function and LA size. However, LA function was decreased in several cases despite absence of LA enlargement (32/72 [44%] with LAD <55 mm; 14/48 [29%] with LAVI <60 mL/m^2^) (Fig. 2). There were also cases in which LA function differed despite equivalent LAVI (Fig. 3).

### Correlations with pulmonary artery systolic pressure during peak exercise

Pulmonary artery systolic pressure (PASP) during peak exercise was correlated with age, resting PASP, LAS-s, LAS-e, LASR-s, LASR-e, and E/E’ during peak exercise. There was no significant correlation between MR severity and PASP either at rest or during peak exercise. Similarly, SV and CO were not associated with PASP during peak exercise (Table 4). Multivariate linear regression analysis indicated that age (coefficient, 0.167; 95% confidence interval [CI], 0.029–0.363, P = 0.031) and LAS-s (coefficient, −0.792; 95% CI, −0.988 to −0.444, P < 0.001) were independent factors associated with PASP during peak exercise (R^2^ = 0.44, P < 0.001) (Table 5). Similar results were found in the multivariable analyses using LASR-s instead of LAS-s. Accordingly, LASR-s was also a statistically significant echocardiographic parameter. However, the measurement of strain rate based on the speckle tracking method has difficulties and poor reproducibility due to relative noise than strain; thus, this study employed strain, as well as the earlier conducted studies, which is clinically useful. Thus, LAS-s was the useful resting echocardiographic parameter that independently predicted PASP during peak exercise.

### LA function predicts EIPH

**Figure 4:**
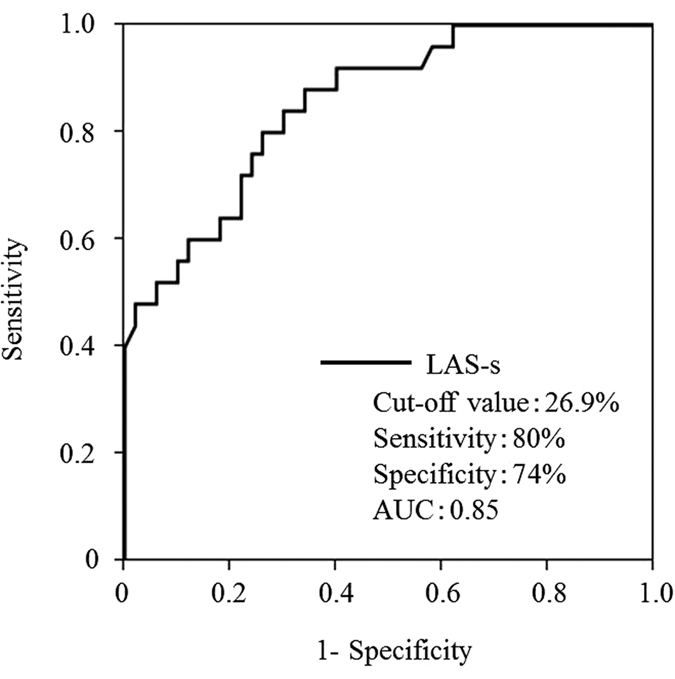
In receiver operating characteristic analysis, left atrial strain (LAS)-s, an index of reservoir function, had an area under the curve (AUC) of 0.85 and could predict exercise-induced pulmonary hypertension with higher accuracy than age at a cut-off value of 26.9% (sensitivity, 80%; specificity, 74%).

In ROC analysis, LAS-s, an index of reservoir function, had an area under the curve of 0.85, and predicted EIPH with higher accuracy than age. Its optimal cut-off value was 26.9% (sensitivity, 80%; specificity, 74%) (Fig. 4).

### Relationship between LA function and prognosis

Follow-up was complete in 75 patients (100%), with a mean follow-up period of 44 ± 21 months. During follow-up, 46 patients (61%) remained asymptomatic and 29 (39%) developed symptoms. Six patients were hospitalized for congestive heart failure, 4 developed new onset atrial fibrillation, and 3 developed resting PH. MV surgery was performed in 22 patients: 3 underwent MV replacement and 19 underwent MV repair. All patients underwent surgery because of symptoms. There was no perioperative mortality.

**Figure 5:**
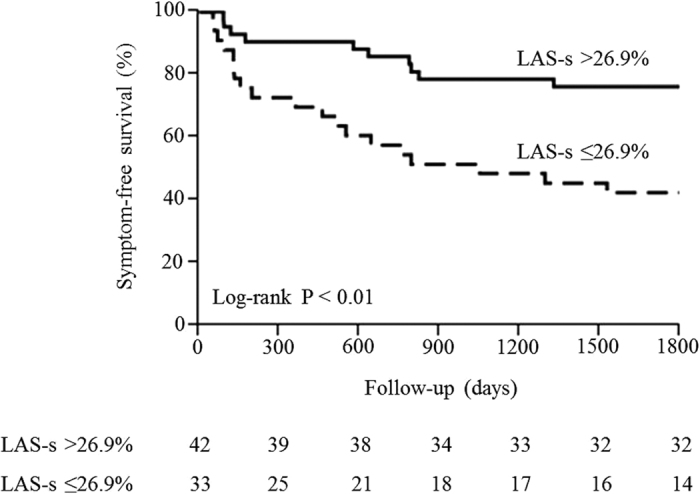
Symptom-free survival according to left atrial strain (LAS)-s, an index of reservoir function.

Based on ROC analysis, all patients were divided into a preserved LAS-s group (LAS-s >26.9%) and a reduced LAS-s group (LAS-s ≤26.9%). During follow-up, symptom-free survival was markedly lower in the reduced LAS-s group (log-rank P < 0.01, Fig. 5). Thus, decreased LA reservoir function was a predictor of EIPH and a useful predictor of prognosis.

## Discussion

The major findings of this study are as follows: First, EIPH occurred in approximately 35% of asymptomatic patients with DMR. Second, LA functional indices using strain analysis are resting indices that can predict EIPH onset. Third, LAS-s, an index of reservoir function, stratified prognosis in asymptomatic DMR.

As aforementioned, the mechanisms of PH onset in MR are manifold^8^. Although this study did not examine pathologic aspects, such as alveolar capillary stress failure, dysfunctional vascular smooth muscle reactivity, and thickening and intimal hyperplasia of the distal pulmonary arterioles, we found that LA function contributed most to EIPH, even more so than LV contractile and diastolic dysfunction. In a previous study, Magne *et al*. demonstrated a relationship between MR severity and EIPH^7^, but there was no such correlation in the current study. This may be because, in comparison to our study, theirs included more severe cases of MR (RV, 62.0 ± 18.1 mL vs. 71 ± 27 mL). We believe this also contributed to the 48-month event-free survival in the EIPH group in their study, which was roughly 15% and lower than the estimated 40% found in our study. Because this study included less severe cases of MR, the decrease in only LAS-s and E/E’ peak exercise in the early stages must have been associated with EIPH, without exhibiting differences associated with MR severity.

LA performance consists of 3 functions: reservoir, conduit, and booster pump^24^. Among these, reservoir dysfunction was most relevant to EIPH. The 2D speckle-tracking method is noninvasive and automatically tracks specific myocardial speckles systemically; it is a new technique that assesses local or global myocardial movement and speed, and its function^25^,^26^,^27^,^28^,^29^. In this study, 2D speckle tracking was applied to the left atrium, and the aforementioned LA functions were each classified and assessed with good reproducibility and accuracy^21^. The reservoir phase begins with ventricular systole, during which the mitral annulus and MV descend because of longitudinal shortening during LV contraction. LA volume increases while LA pressure decreases because of active relaxation of the atrium, and the left atrium receives blood from the pulmonary veins during ventricular systole. In other words, LA reservoir function is mainly determined by LA compliance (stiffness) and LV systolic function (movement of the LV base towards the ventricular apex)^30^,^31^,^32^. However, because all subjects in this study were required to have preserved LVEF, decreased LA reservoir function is considered to have been primarily due to decreased LA compliance rather than LV systolic function. It has been reported that LA reservoir dysfunction observed in patients with DMR is caused by ultrastructural changes in the LA myocardium, such as interstitial fibrosis, myocyte hypertrophy, chronic inflammatory changes, and decreased capillary density^23^,^33^,^34^,^35^,^36^. Because MR jets flow into the left atrium during ventricular systole, the corresponding reservoir function during that moment is critical to patients with MR. The results of the current study also showed that of all LA functions, reservoir function most profoundly contributed to EIPH.

LA enlargement resulting from volume overload in DMR influences severity and duration of MR^37^. The relationship between LAD and mortality in patients with DMR caused by MVP has been demonstrated in the Mitral Regurgitation International Database registry. Cases with LAD ≥55 mm showed extremely low 8-year survival, and this was an independent predictor of all-cause and cardiovascular mortality^38^. Furthermore, Le Tourneau *et al*. showed that LA volume played a role in 5-year survival: in patients with LAVI ≥60 mL/m^2^, mortality and cardiovascular events were reported to increase^39^. However, in our study, we did not observe a significant correlation between EIPH and either LAD or LAVI. The left atrium expands as a compensatory change to volume overload associated with MR; however, in our study, there were several patients who had LAD ≥55 mm (4%) or LAVI ≥60 mL/m^2^ (36%). This may have been due to the patients in our study being younger than those in previous studies, with shorter MR durations, and our study may have included less severe cases of MR (RV, 62.0 ± 18.1 mL vs. 68 ± 42 mL). LAS-s has been reported to decrease from moderate stages of MR^40^; our data confirmed this observation even in moderate cases. Additionally, in a previous study, 47 (48%) of 98 patients with LAD <55 mm and 15 (34%) of 44 patients with LAVI <60 mL/m^2^ demonstrated LA dysfunction^23^, similar to the results obtained in our study. Although there was certainly a correlation between LA function and size in this study, LA reservoir function was considered to acutely influence findings of volume overload in DMR at an earlier stage than LA size because such a decline in LA function began to occur earlier than LA enlargement. Therefore, evaluation of LA function using 2D speckle tracking can be used to predict EIPH in asymptomatic patients with DMR at an earlier stage than LA enlargement.

The present study has several limitations. First, our data were taken from a small sample at a single facility. Second, we did not assess LA function using 2D speckle-tracking during peak exercise. This was because of a technical difficulty: heart rate during peak exercise was too fast, and the LA wall was too thin to track with the software used in this study. Third, right atrial pressure, a fundamental aspect of our study, was not constant among subjects. Additionally, despite the fact that variation was observed even within the same patient depending on his/her condition, right atrial pressure used for PASP calculation in this study was estimated using a semiquantitative method involving diameter and respiratory change of the inferior vena cava. Although this estimation was performed in the same manner at rest and during exercise, it is possible that changes in right atrial pressure during exercise cannot be accurately recorded using this method. As with other studies, use of noninvasive techniques in this study to evaluate right atrial pressure at rest and during exercise has its limitations, and remains a concern.

In conclusion, EIPH occurred in approximately 35% of asymptomatic patients with moderate to advanced DMR. LAS-s, an index of LA reservoir function, was the only resting echocardiographic indicator that independently predicted EIPH. Furthermore, LAS-s was associated with a decrease in symptom-free survival. Based on our results, we propose that evaluation of LA function using 2D speckle tracking is useful for predicting EIPH and prognosis in asymptomatic patients with DMR.

## Additional Information

**How to cite this article**: Kamijima, R. *et al*. Predictors of Exercise-Induced Pulmonary Hypertension in Patients with Asymptomatic Degenerative Mitral Regurgitation: Mechanistic Insights from 2D Speckle Tracking Echocardiography. *Sci. Rep.* **7**, 40008; doi: 10.1038/srep40008 (2017).

**Publisher's note:** Springer Nature remains neutral with regard to jurisdictional claims in published maps and institutional affiliations.

## Figures and Tables

**Table 1:** Baseline characteristics.

	All patients (n = 75)	Non-EIPH (n = 50, 66.7%)	EIPH (n = 25, 33.3%)	P value
**Clinical characteristics**				
Age, y	59.1 ± 13.1	56.6 ± 13.4	64.0 ± 11.2	0.01
Male sex	56 (74.7)	37 (74.0)	19 (76.0)	0.85
BMI, kg/m^2^	22.2 ± 3.2	21.9 ± 3.3	22.7 ± 3.2	0.33
Heart rate, bpm	69.5 ± 10.9	69.0 ± 11.5	70.6 ± 9.8	0.54
SBP, mmHg	132.7 ± 17.1	130.6 ± 14.3	136.9 ± 21.3	0.16
DBP, mmHg	74.3 ± 10.1	74.0 ± 9.8	74.7 ± 10.8	0.72
Log BNP, pg/mL	1.49 ± 0.32	1.46 ± 0.29	1.53 ± 0.37	0.31
**Risk factors**				
Hypertension	31 (41.3)	16 (32.0)	15 (60.0)	0.02
Dyslipidemia	14 (18.7)	10 (20.0)	4 (16.0)	0.68
Diabetes mellitus	0 (0)	0 (0)	0 (0)	-
**Medications**				
ACE-I/ARB	20 (26.7)	10 (20.0)	10 (40.0)	0.06
β-blocker	6 (8.0)	2 (4.0)	4 (16.0)	0.07
Calcium channel blocker	17 (22.7)	6 (12.0)	11 (44.0)	0.002
Diuretic	13 (17.3)	6 (12.0)	7 (28.0)	0.08
Statin	10 (13.3)	7 (14.0)	3 (12.0)	0.81
**Etiology of mitral regurgitation**				
Anterior leaflet prolapse	22 (29.3)	16 (32.0)	6 (24.0)	0.47
Posterior leaflet prolapse	48 (64.0)	31 (62.0)	17 (68.0)	0.61
Both leaflet prolapse	2 (2.7)	1 (2.0)	1 (4.0)	0.61
Sclerotic change	3 (4.0)	2 (4.0)	1 (4.0)	1.00

Data are expressed as mean ± SD or number (percentage), as appropriate.

Abbreviations: ACE-I, angiotensin-converting enzyme inhibitor; ARB, angiotensin receptor blocker; BMI, body mass index; BNP, brain natriuretic peptide; DBP, diastolic blood pressure; EIPH, exercise-induced pulmonary hypertension; SBP, systolic blood pressure.

**Table 2:** Resting and exercise echocardiographic data.

	All patients (n = 75)	Non-EIPH (n = 50, 66.7%)	EIPH (n = 25, 33.3%)	P value
**Resting**				
LVESVI, mL/m^2^	27.0 ± 6.4	27.9 ± 6.7	25.4 ± 5.6	0.14
LVEDVI, mL/m^2^	81.1 ± 17.8	82.1 ± 18.3	79.1 ± 17.0	0.54
LVEF, %	67.6 ± 4.7	67.1 ± 4.7	68.6 ± 4.6	0.16
E-wave velocity, cm/s	105.5 ± 27.4	101.2 ± 24.4	114.2 ± 31.3	0.11
A-wave velocity, cm/s	67.2 ± 16.9	64.9 ± 16.2	72.0 ± 17.6	0.07
E/A ratio	1.66 ± 0.60	1.69 ± 0.62	1.59 ± 0.56	0.48
E/E’	11.5 ± 3.6	11.1 ± 3.4	12.4 ± 3.9	0.09
LAVI, mL/m^2^	54.2 ± 18.9	52.8 ± 18.9	56.9 ± 18.8	0.36
Severe MR	35 (46.7)	22 (44.0)	13 (52.0)	0.51
EROA, mm^2^	0.41 ± 0.12	0.41 ± 0.13	0.42 ± 0.11	0.57
Regurgitant volume, mL	62.0 ± 18.1	61.6 ± 19.6	62.9 ± 14.8	0.53
PASP, mmHg	28.8 ± 4.4	28.1 ± 4.1	30.1 ± 4.7	0.09
Stroke volume, mL	59.0 ± 12.4	60.2 ± 12.0	56.7 ± 13.0	0.28
Cardiac output, L	4.05 ± 0.90	4.10 ± 0.97	3.94 ± 0.77	0.65
**Peak exercise**				
LVESVI, mL/m^2^	24.8 ± 6.3	24.5 ± 6.0	25.4 ± 7.0	0.88
LVEDVI, mL/m^2^	84.0 ± 16.5	82.2 ± 18.0	85.4 ± 13.3	0.58
LVEF, %	70.6 ± 5.0	70.3 ± 4.9	71.3 ± 5.2	0.45
E-wave velocity, cm/s	144.0 ± 27.1	140.2 ± 28.8	151.4 ± 22.1	0.11
A-wave velocity, cm/s	110.9 ± 25.8	108.1 ± 27.9	116.6 ± 20.2	0.34
E/A ratio	1.36 ± 0.45	1.35 ± 0.50	1.38 ± 0.27	0.40
E/E’	12.0 ± 4.0	11.1 ± 3.4	13.7 ± 4.6	0.01
LAVI, mL/m^2^	49.5 ± 17.8	48.4 ± 17.9	51.8 ± 17.8	0.43
Severe MR	58 (77.3)	36 (72.0)	22 (88.0)	0.12
EROA, mm^2^	0.49 ± 0.17	0.48 ± 0.18	0.51 ± 0.16	0.21
Regurgitant volume, mL	65.9 ± 15.9	63.9 ± 18.0	66.3 ± 10.5	0.09
PASP, mmHg	48.8 ± 12.5	41.4 ± 8.2	63.3 ± 3.6	<0.001
Stroke volume, mL	62.6 ± 14.1	64.3 ± 13.7	59.3 ± 14.5	0.20
Cardiac output, L	7.71 ± 1.94	7.77 ± 1.83	7.59 ± 2.19	0.80
**Delta (peak exercise – resting)**				
ΔEROA, mm^2^	0.11 ± 0.13	0.09 ± 0.14	0.13 ± 0.08	0.06
ΔRegurgitant volume, mL	6.0 ± 14.2	4.9 ± 15.3	8.2 ± 11.9	0.35
ΔStroke volume, mL	3.5 ± 9.3	3.9 ± 10.1	2.6 ± 7.8	0.96
ΔCardiac output, L	3.66 ± 1.6	3.66 ± 1.54	3.65 ± 1.77	0.99

Data are expressed as mean ± SD or number (percentage), as appropriate.

Abbreviations: EIPH, exercise-induced pulmonary hypertension; EROA, effective regurgitant orifice area; LAVI, left atrial volume index; LVEDVI, left ventricular end-diastolic volume index; LVEF, left ventricular ejection fraction; LVESVI, left ventricular end-systolic volume index; MR, mitral regurgitation; PASP, pulmonary artery systolic pressure.

**Table 3:** Echocardiographic data on left atrial volume and function at rest.

	All patients (n = 75)	Non-EIPH (n = 50, 66.7%)	EIPH (n = 25, 33.3%)	P value
LAD, mm	40.9 ± 6.9	39.9 ± 7.2	42.7 ± 5.9	0.08
LAVI, mL/m^2^	54.2 ± 18.9	52.8 ± 18.9	56.9 ± 18.8	0.36
LAS-s, %	29.2 ± 7.8	32.4 ± 6.8	22.9 ± 5.5	<0.001
LAS-e, %	18.2 ± 6.8	19.9 ± 6.8	14.8 ± 5.4	0.003
LAS-a, %	10.9 ± 4.8	11.7 ± 5.0	9.3 ± 4.1	0.05
LASR-s, s^−1^	1.32 ± 0.34	1.42 ± 0.33	1.11 ± 0.26	<0.001
LASR-e, s^−1^	1.29 ± 0.36	1.36 ± 0.36	1.13 ± 0.29	0.003
LASR-a, s^−1^	1.30 ± 0.35	1.35 ± 0.39	1.18 ± 0.23	0.07

Data are expressed as mean ± SD.

Abbreviations: EIPH, exercise-induced pulmonary hypertension; LAD, left atrial diameter; LAS, left atrial strain; LASR, left atrial strain rate; LAVI, left atrial volume index.

**Table 4:** Correlations with PASP during peak exercise.

Variable	Correlation with PASP during peak exercise	
	R	P value
Age	0.38	<0.001
**Resting**		
Heart rate	0.03	0.78
SBP	0.04	0.76
DBP	0.13	0.28
LVESVI	0.18	0.12
LVEDVI	0.14	0.25
LVEF	0.21	0.07
E/A ratio	0.12	0.30
E/E’	0.23	0.05
EROA	0.03	0.78
Regurgitant volume	0.04	0.71
PASP	0.25	0.03
Stroke volume	0.16	0.17
Cardiac output	0.19	0.11
LAD	0.22	0.06
LAVI	0.21	0.07
LAS-s	−0.58	<0.001
LAS-e	−0.38	<0.001
LAS-a	−0.25	0.03
LASR-s	−0.47	<0.001
LASR-e	−0.39	0.001
LASR-a	−0.23	0.05
**Peak exercise**		
Heart rate	0.18	0.13
SBP	0.16	0.17
DBP	0.09	0.46
LVESVI	0.01	0.91
LVEDVI	0.07	0.57
LVEF	0.20	0.09
E/A ratio	0.05	0.68
E/E’	0.31	0.01
EROA	0.10	0.40
Regurgitant volume	0.20	0.09
LAD	0.17	0.14
LAVI	0.15	0.21
Stroke volume	0.20	0.08
Cardiac output	0.10	0.42
**Delta (peak exercise – resting)**		
ΔEROA	0.14	0.22
ΔRegurgitant volume	0.14	0.23
ΔStroke volume	0.09	0.47
ΔCardiac output	0.01	0.93

Abbreviations: DBP, diastolic blood pressure; EROA, effective regurgitant orifice area; LAD, left atrial diameter; LAS, left atrial strain; LASR, left atrial strain rate; LAVI, left atrial volume index; LVEDVI, left ventricular end-diastolic volume index; LVEF, left ventricular ejection fraction; LVESVI, left ventricular end-systolic volume index; PASP, pulmonary artery systolic pressure; SBP, systolic blood pressure.

**Table 5:** Multiple linear regression analysis to determine PASP during peak exercise.

Variable	Coefficient	95% CI	P value
Age	0.167	0.029 to 0.363	0.031
Resting PASP	0.187	−0.385 to 0.760	0.516
LAS-s	−0.792	−0.988 to −0.444	<0.001
Peak exercise E/E’	0.526	−0.079 to 1.130	0.097
ΔEROA	7.572	−12.476 to 27.619	0.454
ΔStroke volume	0.070	−0.333 to 0.193	0.596

Abbreviations: CI, confidence interval; EROA, effective regurgitant orifice area; LAS, left atrial strain; PASP, pulmonary artery systolic pressure.
